# A case of osteophyte excision and arthroscopic arthrodesis for tarsal tunnel syndrome with traumatic osteoarthritis of the ankle

**DOI:** 10.1016/j.ijscr.2020.10.053

**Published:** 2020-10-21

**Authors:** Ichiro Tonogai, Koichi Sairyo

**Affiliations:** Department of Orthopedics, Institute of Biomedical Science, Tokushima University Graduate School, 3-18-15 Kuramoto, Tokushima City, Tokushima, 770-8503, Japan

**Keywords:** Tarsal tunnel syndrome, Osteophyte, Osteoarthritis, Arthroscopic arthrodesis, Ankle

## Abstract

**Introduction:**

There are some reports of tarsal tunnel syndrome (TTS) entrapment/impingement from bony factors, including exostosis and fragment, but there are no reports on TTS with traumatic osteoarthritis of the ankle that were treated with osteophyte excision for TTS and arthroscopic arthrodesis for osteoarthritis of the ankle.

**Presentation of case:**

A 61-year-old woman with left trimalleolar fracture had undergone surgery 3 years earlier and was referred to our hospital for further investigation of persistent left ankle pain and numbness around the left medial malleolus and plantar aspect of the foot. Clinical examination demonstrated plantar hypesthesia and a positive Tinel’s sign at the tarsal tunnel. Imaging showed severe osteoarthritic change in the ankle and an osteophyte of the posteromedial distal tibia that appeared to be impinging on the tibial nerve. We performed arthroscopic ankle arthrodesis, which is less invasive than the open procedure, with removal of the osteophyte as the cause of TTS. Tarsal tunnel exploration revealed a large osteophyte pushing on the tibial nerve, and the osteophyte was removed.

**Discussion:**

About 8 weeks after surgery, bony union was achieved. At the 2-year follow-up visit, the patient could perform daily activities with almost no pain or numbness. This case offers further insight into the management of TTS with traumatic osteoarthritis of the ankle.

**Conclusion:**

We report here successful treatment of a rare case of tarsal tunnel syndrome (TTS) accompanied with traumatic osteoarthritis of the ankle, treated with osteophyte excision for the TTS and arthroscopic for the osteoarthritis.

## Highlights

• We successfully treated tarsal tunnel syndrome (TTS) accompanied with traumatic osteoarthritis of the ankle.
• Osteophyte excision for the TTS and arthroscopic for the osteoarthritis was effective for even TTS with traumatic osteoarthritis of the ankle.
• There were many advantages in arthroscopic ankle arthrodesis, compared with open ankle arthrodesis.

## 1 Introduction

The ankle is a congruent, generally stable joint that transmits high peak contact stress across a very thin layer of articular cartilage [1]. Because any change in the congruence of the ankle can lead to an increase in the forces across the ankle and accelerated degeneration, ankle osteoarthritis is relatively common and is predominantly related to previous trauma [2], occurring after trauma in 78 % of cases [3]. Osteophytes are commonly observed in osteoarthritis.

The posterior tibial neurovascular complex is tightly constrained in the tarsal tunnel because the contents are often attached to the fibrous septa within the tunnel [4,5]. Therefore, tarsal tunnel syndrome (TTS) can be caused by entrapment resulting from space-occupying lesions, including varicosity of the posterior tibial veins, ganglion, synovial cyst, aneurysm, neurofibroma, neurilemmoma, lipoma, rheumatoid nodule, bony exostosis, hypertrophy of the abductor hallucis muscle, and tumor [6]. Clinical signs include sensory disturbance, muscle weakness over the distribution of the posterior tibial nerve or its terminal branches, and a positive Tinel’s sign. There are some reports of TTS entrapment/impingement from bony factors, including exostosis and fragment [6,7]. However, to our knowledge there are no reports on TTS with traumatic osteoarthritis of the ankle that were treated with osteophyte excision for TTS and arthroscopic arthrodesis for osteoarthritis of the ankle. We report here such a rare case of TTS accompanied with traumatic osteoarthritis of the ankle, treated with osteophyte excision for the TTS and arthroscopic arthrodesis for the osteoarthritis.

This has been reported in line with the SCARE criteria [8].

## 2 Presentation of case

The patient granted permission for the publication of this case report.

A 61-year-old woman was referred to our hospital 3 years after undergoing surgery for a left trimalleolar fracture of the ankle due to a road traffic accident. She underwent immediate open reduction and internal fixation at a local hospital. Left ankle pain persisted and about 10 months after the primary surgery she developed numbness along the medial aspect of the left ankle and over the sole of the foot. The implants were removed at about 1 year after the primary surgery, but the left ankle pain did not improve and the numbness worsened. She was referred to us for further investigation.

**Fig. 1:**
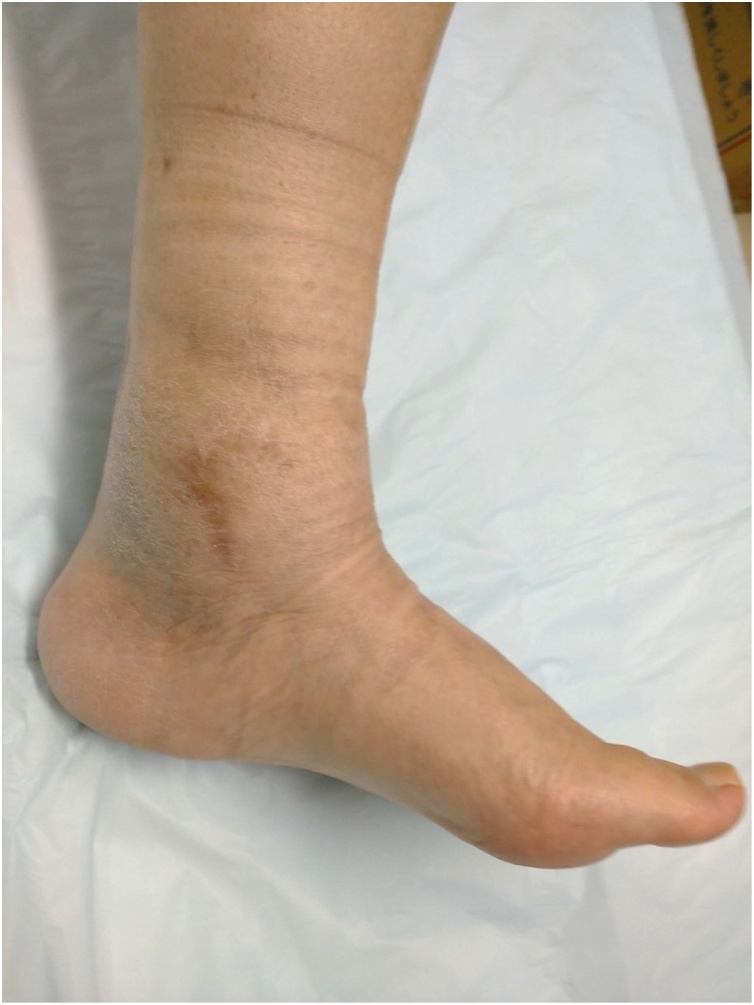
Photograph showing swelling over the proximal posterior aspect of the medial malleolus.

**Fig. 2:**
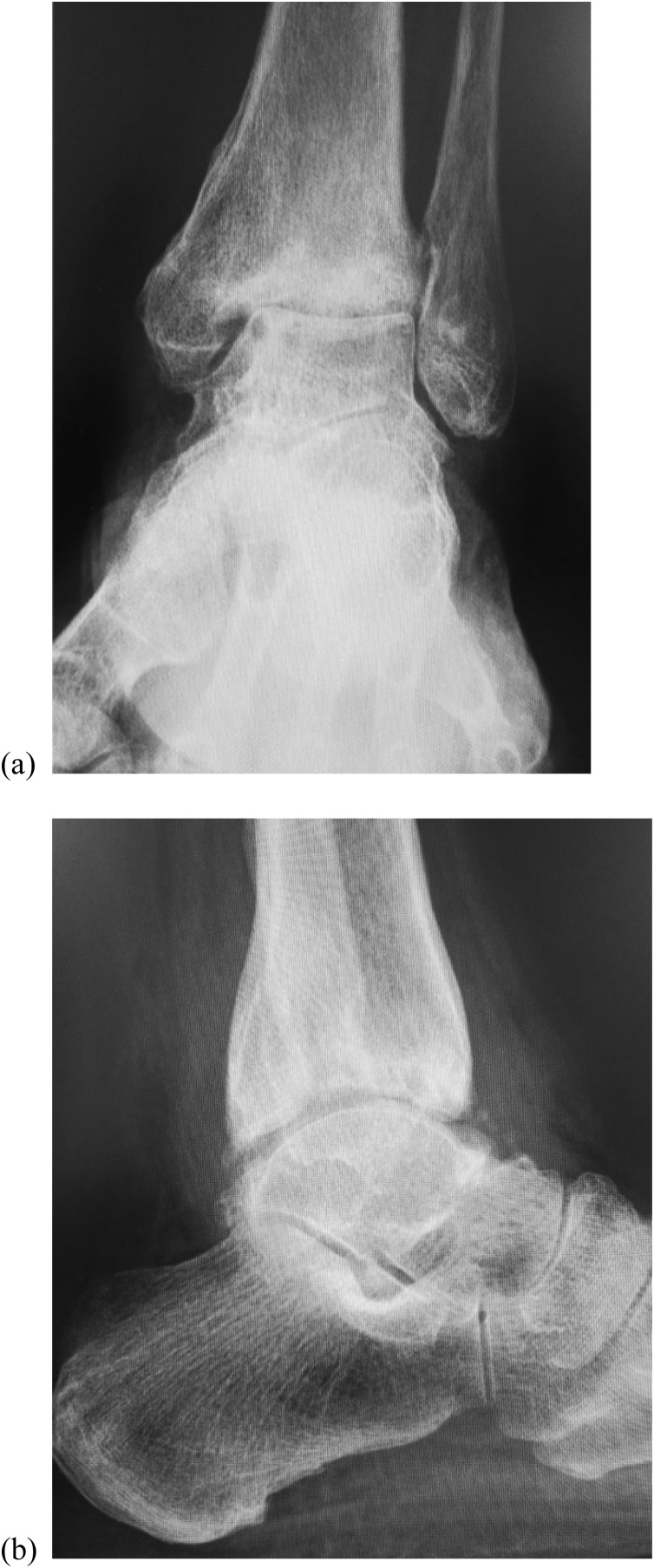
Preoperative plain radiography images. Images showing severe tibiotalar joint space narrowing, indicating osteoarthritic change in the standing position on (a) anterior-posterior view and (b) lateral view.

**Fig. 3:**
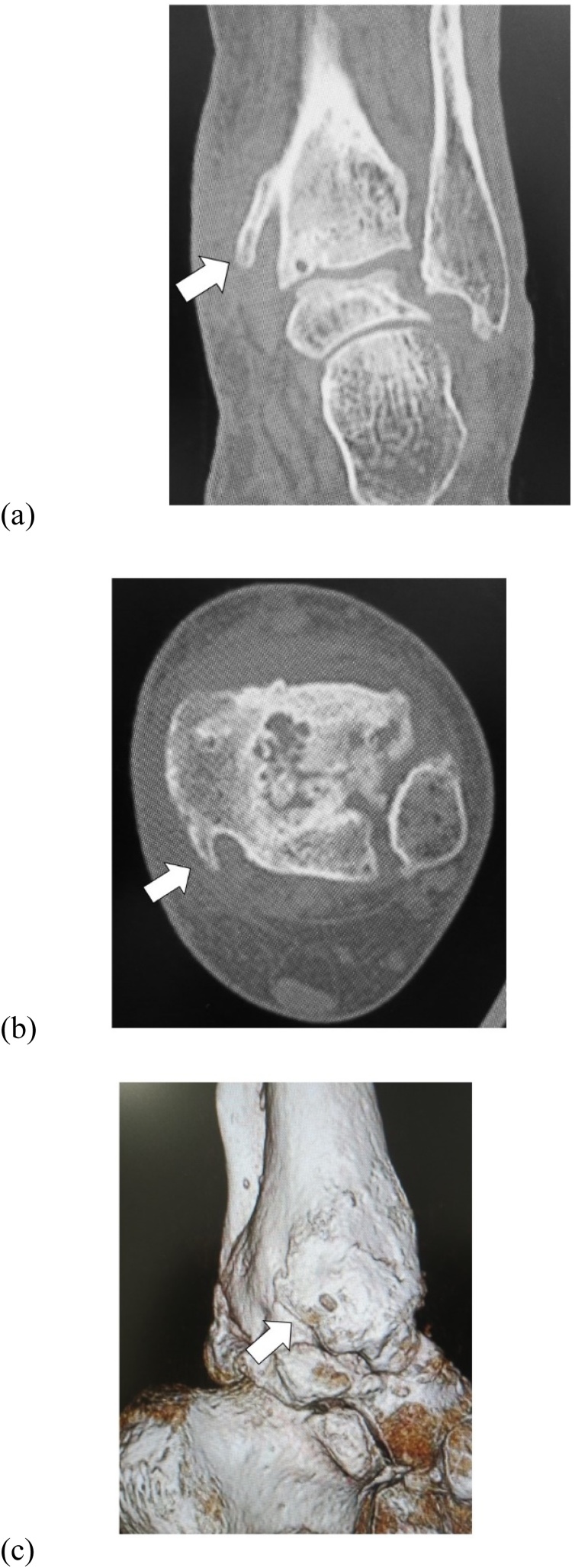
Preoperative computed tomography (CT) images. CT shows osteophyte of the posteromedial aspect of the distal tibia (arrow) on (a) coronal view and (b) axial view and on (c) 3D CT imaging.

**Fig. 4:**
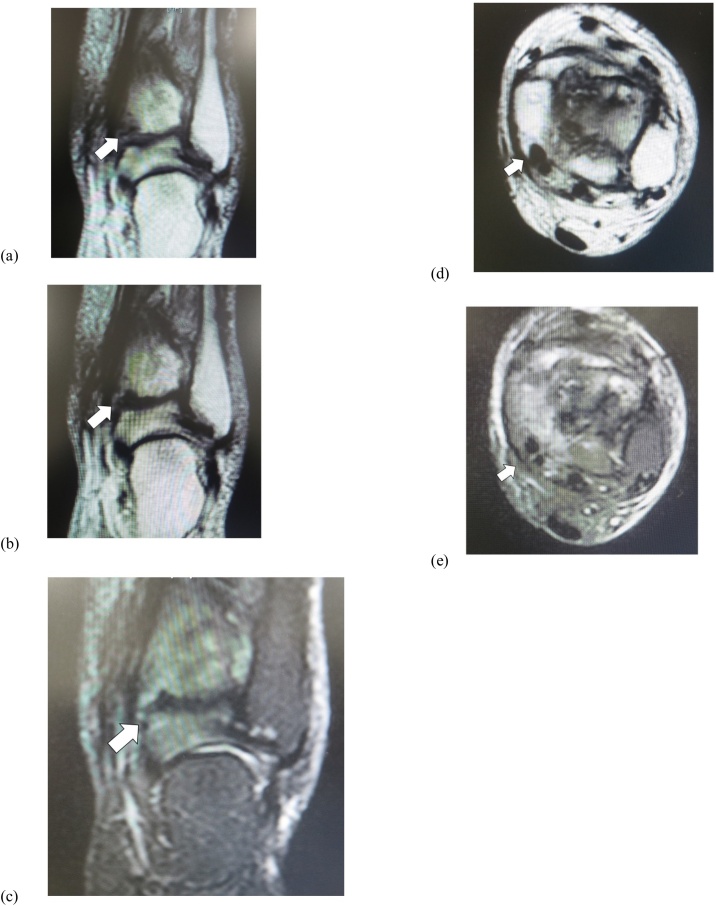
Preoperative magnetic resonance imaging (MRI) findings. MRI shows osteophyte impinging on the tibial nerve (arrow) on (a) T1-weighted, (b) T2-weighted, and (c) short T1 inversion recovery (STIR) images on coronal view, and on (d) T2 and (e) STIR images on axial view.

Her main complaint was a tingling/reduced sensation and paresthesia on the plantar and medial aspects of the forefoot to the middle foot area along the main distribution of the medial plantar nerve. There was tenderness and swelling over the ankle joint and the proximal posterior aspect of the medial malleolus (Fig. 1). Tinel’s-sign was positive over the proximal posterior aspect of the medial malleolus. No motor deficit or deformity of the toes was detected. Plain radiographs revealed severe joint space narrowing between the tibia and talus, indicating osteoarthritic change in the left ankle in the standing position on anterior-posterior view (Fig. 2a) and lateral view (Fig. 2b). Computed tomography (CT) images showed an osteophyte of the posteromedial aspect of the distal tibia on coronal view (Fig. 3a) and axial view (Fig. 3b) and on three-dimensional (3D) CT imaging (Fig. 3c). Magnetic resonance (MR) images showed the osteophyte impinging on the tibial nerve on T1-weighted (Fig. 4a), T2-weighted (Fig. 4b), and short T1 inversion recovery (STIR) images (Fig. 4c) on coronal view, and on T2 (Fig. 4d) and STIR (Fig. 4e) images on axial view.

We diagnosed TTS with traumatic osteoarthritis of the ankle. Therefore, we opted to perform excision of the osteophyte to decompress the tibial nerve and ankle arthroscopic arthrodesis because endoscopic techniques can accelerate recovery and reduce morbidity compared with the open procedure. The preoperative Japanese Society for Surgery of the Foot (JSSF) score was 30/100 points (pain 0/40, function 20/50, alignment 10/10).

**Fig. 5:**
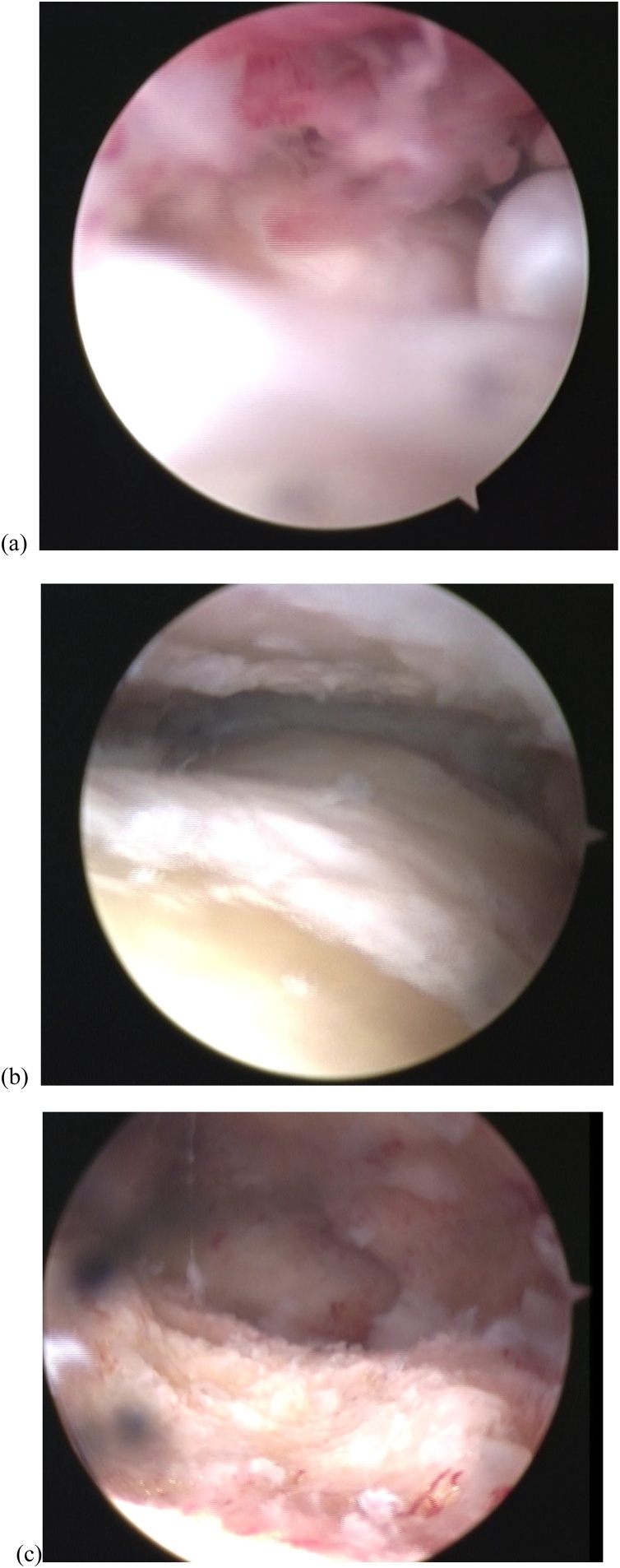
Operative arthroscopy findings. (a) Fibrous tissue is seen filling the ankle joint space. (b) Cartilage is almost gone and subchondral bone is exposed at the articular surface of the tibial plafond and talar trochlea. (c) Using a surgical abrader, all remaining articular cartilage is denuded and beds of bleeding cancellous bone are established.

**Fig. 6:**
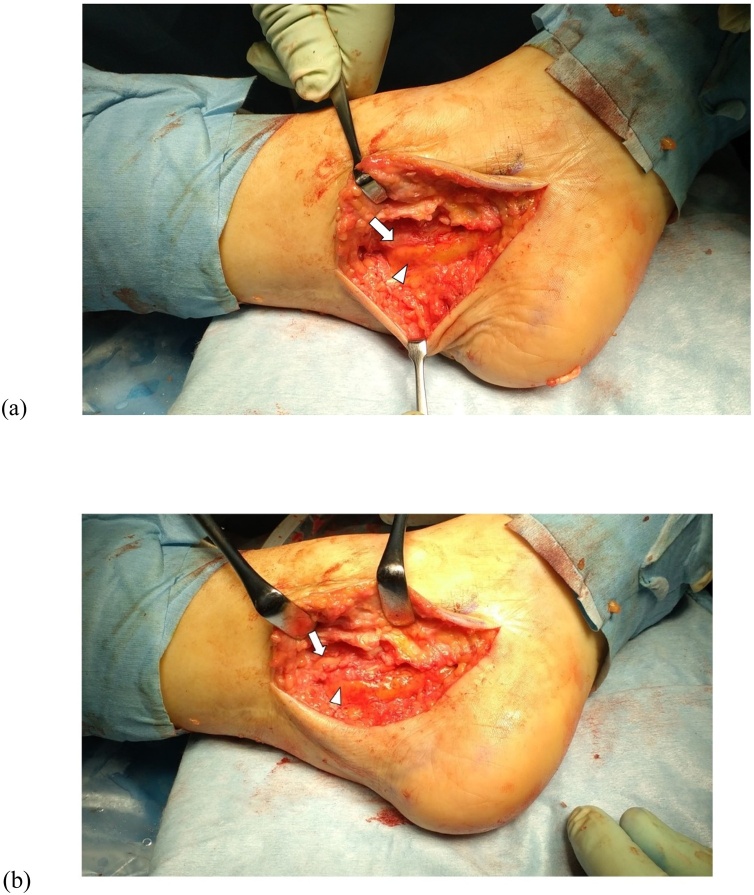
Intraoperative photograph. (a) Osteophyte of the posteromedial aspect of the distal tibia (arrow) is seen impinging on the medial plantar nerve (arrowhead). (b) The osteophyte is excised (arrow) and the tibial nerve released (arrowhead).

**Fig. 7:**
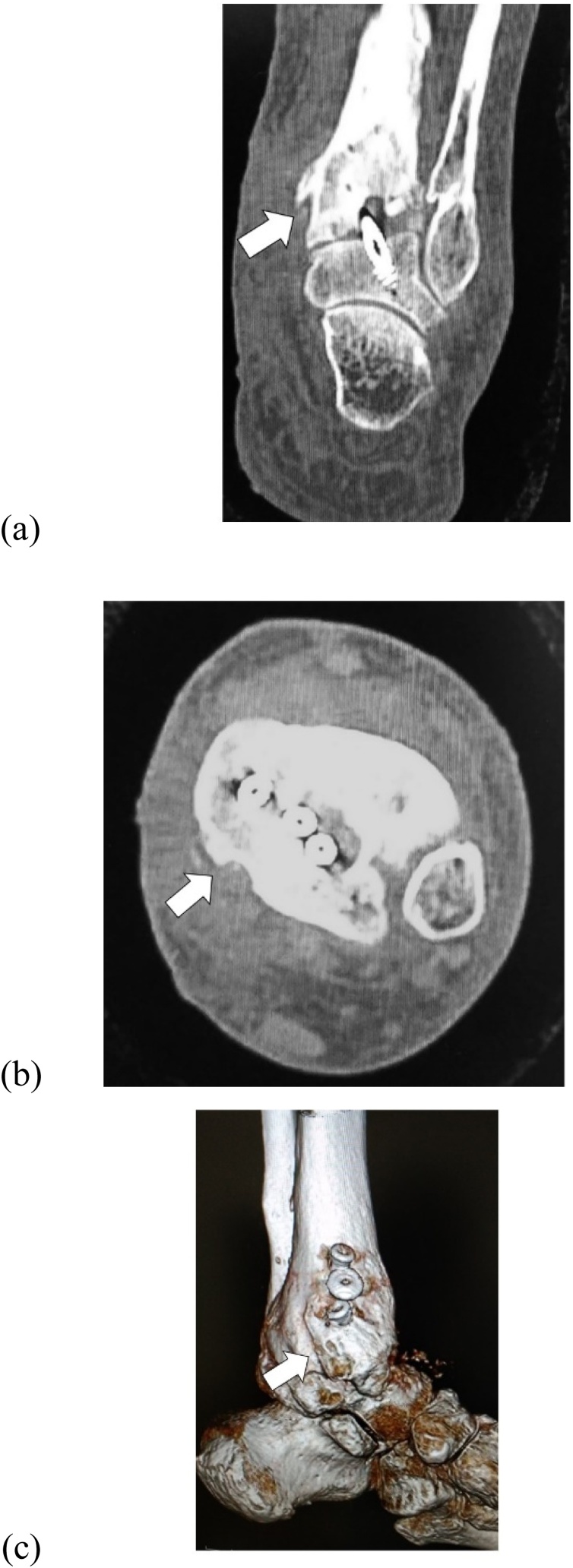
Postoperative computed tomography (CT) images. Osteophyte removal (arrow) on (a) axial, (b) coronal, and (c) 3-DCT images.

The patient was positioned supine with the affected limb in a stirrup and traction in place. Two standard portals (anterolateral and anteromedial) were used. Fibrous tissue was seen filling the ankle joint space (Fig. 5a) and synovitis was severe. The fibrous tissue was removed and synovectomy was performed with an arthroscopic shaver. Cartilage was almost completely denuded and subchondral bone was exposed at the articular surface of the tibial plafond and talar trochlea (Fig. 5b). Using a surgical abrader, we denuded all remaining articular cartilage and established beds of bleeding cancellous bone (Fig. 5c). A curvilinear incision was then placed along the course of the tibial nerve posterior to the medial malleolus. Fixation between the distal tibia and talus was established using 3 cannulated partially threaded screws. After fixation, the flexor retinaculum was released, exposing the osteophyte in the posteromedial side of the distal tibia pushing the tibial nerve from the anterior aspect (Fig. 6a). Scarring around the nerve was also noted with redness and swelling of the nerve. The osteophyte was removed (Fig. 6b). CT images soon after surgery confirmed successful removal of the osteophyte on coronal view (Fig. 7a) and axial view (Fig. 7b) and on a 3D CT image (Fig. 7c).

A non-weightbearing below-knee cast was applied for 2 weeks for immobilization. This immobilization was maintained for another 2 weeks but with weightbearing permitted. After a total of 4 weeks of immobilization with the lower leg cast, the cast was removed and an ankle brace was attached to the left foot and ankle. The patient started a mobilization protocol with progressive passive and active range of motion exercises at 4 weeks after surgery. Bony union of the ankle was achieved about 6 weeks after surgery.

**Fig. 8:**
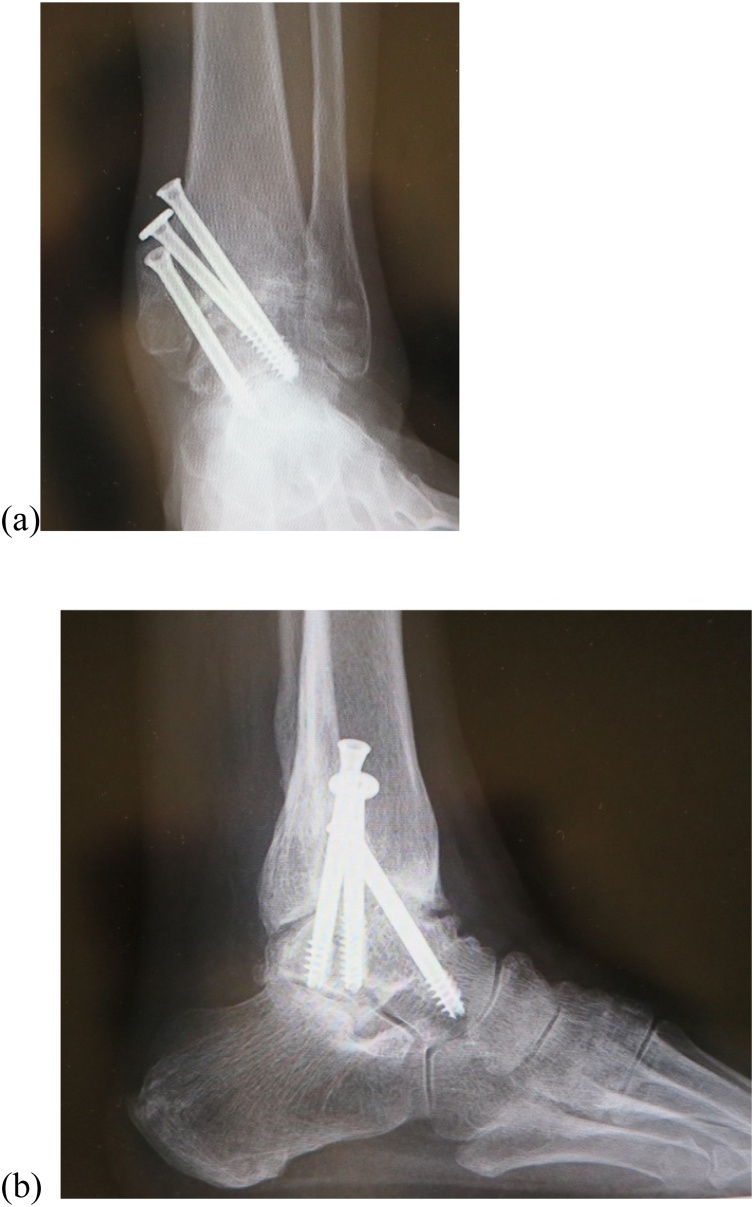
Radiographs 2 years after surgery. Bony union was achieved, seen in the standing position on (a) anteroposterior view and (b) lateral view.

**Fig. 9:**
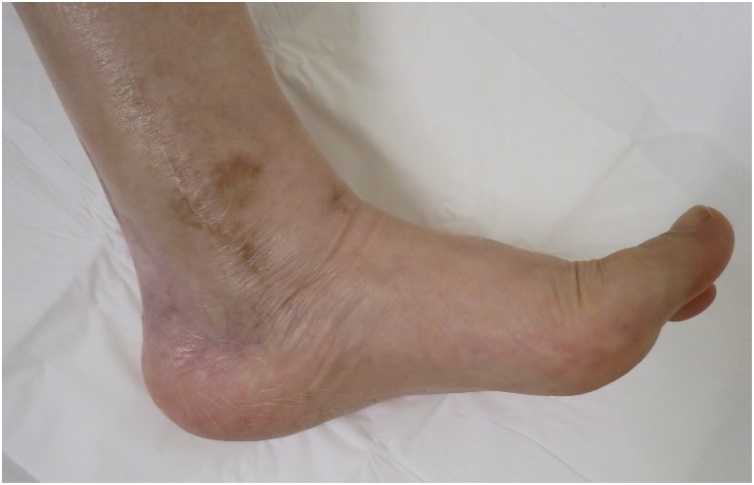
Photograph 2 years after surgery. No swelling is seen over the ankle joint or the proximal posterior aspect of the medial malleolus.

Two years after decompression of the right tarsal tunnel and neurolysis of the tibial nerve, the patient reported major improvements in the dysesthesia along the entire plantar surface of the foot. She was not taking any medication, as radiographs showed complete union between the tibia and the talus on weightbearing on antero-posterior view (Fig. 8a) and lateral view (Fig. 8b). There was no tenderness or swelling over the ankle joint or the proximal posterior level of the medial malleolus (Fig. 9). Tinel’s sign was negative over the proximal posterior level of the medial malleolus. At that time, The JSSF score had improved to 89/100 points (pain 40/40, function 39/50, alignment 10/10).

## 3 Discussion

We have described our management of a 61-year-old woman who presented with TTS with traumatic osteoarthritis of the ankle, for whom osteophyte excision for TTS and arthroscopic arthrodesis for osteoarthritis of the ankle was successful. Regarding TTS entrapment/impingement from bony factors, Bejjanki et al. reported on a case of TTS following ankle replacement surgery secondary to a large displaced osteophyte [7]. Hong also reported on a case of successful treatment of TTS caused by os sustentaculum [9]. However, to our knowledge, there is the first report of TTS with osteophyte due to traumatic osteoarthritis of the ankle treated with osteophyte excision and arthroscopic arthrodesis.

In severe cases of TTS, deformity of the toes can occur due to contracture of the intrinsic muscles of the foot, but there was no toe deformity in our case. There is no consensus yet on the timing of intervention, but some authors suggest that nerve recovery is poor when decompression is delayed beyond 10–12 months [5]. In our case, further delay in releasing the tarsal tunnel might have caused poor nerve recovery because of the osteophyte firmly impinging on the tibial nerve.

Regarding treatment of TTS, Day and Naples described their procedure of endoscopic tarsal tunnel decompression for 5 cases, all with excellent results [10]. Krishnan et al. also described their endoscopic technique for treating patients with TTS as evaluated in a clinical trial [11]. Also, a clinical study by El Shazly et al. evaluated the anatomical basis, safety, and outcomes of endoscopic tarsal tunnel release using a modified trocar cannula and a dilator system [12]. While we performed an open release of the tarsal tunnel and not the endoscopic procedure, the latter might have been preferable for TTS considering the advantage of limited soft tissue trauma and therefore faster recovery.

As to the reason for occurrence, osteoarthritis of the ankle could have been attributed to the posterior malleolar fragment, which Macko et al. suggested was important in the development of post-traumatic osteoarthritis [13]. Our case seems to support their suggestion, because the patient had trimalleolar fracture, as well as posterior malleolar fracture. This might have accelerated the progression of the ankle osteoarthritis compared with the typical progression of ankle osteoarthritis.

In the surgical treatment of ankle osteoarthritis, arthroscopic ankle arthrodesis has gained in popularity in recent years. This procedure has been recommended in end-stage arthritis, mostly in osteo-, rheumatoid, and posttraumatic arthritis [[14], [15], [16], [17]] after failed conservative management. The high rate of bony union is probably due to the limited exposure and significantly decreased periosteal stripping, which probably enhances the process of fusion and facilitates quicker union [[18], [19], [20]]. In light of this, we selected arthroscopic arthrodesis of the ankle, not open arthrodesis of the ankle, although a meticulous surgical technique involving limited soft tissue dissection and diminished devascularization of the bone is key to successful arthrodesis.

A limitation of this report is the short follow-up duration. At the 2-year follow-up after surgery, the patient had almost no ankle pain or numbness; nevertheless, further follow-up is necessary because of possible recurrence of the TTS. Also, the loss of ankle motion caused by arthrodesis is a limitation to consider. The procedure increases strain on the small joints of the ipsilateral foot, and many patients develop degenerative changes in the subtalar and midtarsal joints [21,22]. However, ankle fusion is a well-established procedure with few complications and good pain relief that improves walking ability [23].

## 4 Conclusion

We encountered a rare case of TTS with traumatic osteoarthritis of the ankle, successfully treated with osteophyte excision for TTS and arthroscopic arthrodesis for ankle osteoarthritis. This suggests the utility of arthroscopic arthrodesis as a less invasive and effective procedure even for osteoarthritis of the ankle with TTS.

## Declaration of Competing Interest

The authors report no declarations of interest.

## Funding

This research received no specific grant from any funding agency in the public, commercial, or not-for-profit sectors.

## Ethical approval

A clinical case report is exempt from ethical approval in our institution.

## Consent

A written informed consent was obtained from the patient for publication of this case report and accompanying images. A copy of the written consent is available for review by the Editor-in-Chief of this journal on request.

## Author contribution

Ichiro Tonogai: data collection, writing the paper.

Koichi Sairyo: Interpretation.

## Registration of research studies

None.

## Guarantor

Ichiro Tonogai: i.tonogai@tokushima-u.ac.jp.

Koichi Sairyo: sairyokun@hotmai.com.

## Provenance and peer review

Not commissioned, externally peer-reviewed.
